# Preferable location of chromosomes 1, 29, and X in bovine spermatozoa

**DOI:** 10.3934/genet.2018.2.113

**Published:** 2018-03-21

**Authors:** Vadim Chagin, Andrei Zalensky, Igor Nazarov, Olga Mudrak

**Affiliations:** 1 The Jones Institute for Reproductive Medicine, Eastern Virginia Medical School, Virginia, USA; 2 Institute of Cytology, Russian Academy of Sciences, St. Petersburg, Russian Federation

**Keywords:** chromosome positioning, chromosome territories, sperm nucleus, genome architecture, bovine spermatozoa

## Abstract

Chromosome positioning in sperm nucleus may have a functional significance by influencing the sequence of post-fertilization events. In this study we present data on preferential locations of chromosomes 1, 29 and X in *Bos taurus* spermatozoa. Here we demonstrate that the position of X chromosome in the sperm nucleus is more restricted as compared to the position of chromosome 1, which is about of the same size. Our data support the concept of the functional significance of genome architecture in male germline cells.

## 1. Introduction

Higher order nuclear genome architecture in somatic cells has been shown to be a crucial epigenetic factor that is involved in the regulation of gene expression and influences various aspects of nuclear metabolism. It was suggested that the genome architecture of male gametes may affect the pattern and sequence of paternal gene expression upon fertilization and, therefore, possess a functional significance [1]–[3]. In line with that, fertility associated changes in chromosomes positions and sperm nuclear architecture have been investigated [4]–[8].

Similarly to somatic cells, organization of chromosomes in the distinct non-randomly positioned territories (“chromosome territories” see Cremer T, Cremer M (2010), [9], for review) has been demonstrated by comprehensive studies of chromatin organization in human spermatozoa [10]–[16]. Such non-random genome architecture in mammalian spermatozoa appears to be evolutionary conserved as evidenced from studies of chromosome positioning in spermatozoa of marsupials and monotremes—non-placental mammals evolutionary diverged from placental ones 170 and 130 million years ago, respectively [1],[2],[17], and such placental mammals as rats [18], pigs [19], and humans [10]–[15].

The sperm genome architecture is established during spermatogenesis, which is accompanied by intranuclear chromatin rearrangement. This rearrangement is manifested by changes in positions of individual chromosomes as well as of particular chromosomal regions [18]–[23].

For example, in mammalian primary spermatocytes, both sex chromosomes are located at the nuclear periphery, where they form the so-called XY body [21]. However, in the course of spermatogenesis, X- and Y-chromosomes relocate to the interior of the sperm nucleus by the spermatid stage and retain this position in mature spermatozoa in pigs [19], mice [23], and humans [14].

Changes in the intranuclear positioning in the course of spermatogenesis were also observed for autosomes [19]. These changes seem to be specific to particular chromosomes, as demonstrated by migration of chromosome 13 from the nuclear interior to the periphery between the secondary spermatocyte and round spermatid stage in pigs, where the position of chromosome 5 remained stable [19]. In support to the concept of global spermatogenesis-associated chromatin rearrangement, dynamic repositioning of telomeres [18],[20],[22], clustering of individual centromere domains as well as formation of chromocenters in the nuclear interior (by the round spermatid stage) were reported in rats [18], humans [20], and mice [23].

The global chromosome arrangement in spermatozoa of livestock species was studied only in pigs [19]. Studies of bovine male gametes were limited to identification of sex chromosomes for the selection of male/female spermatozoa [24],[25].

In this work, using Fluorescence in Situ Hybridization (FISH) with whole chromosome painting (WCP) probes and statistical image analysis [14], we investigated intranuclear locations of the largest *Bos taurus* (chromosome 1, BTA1) and the smallest (chromosome 29, (BTA29) autosomes and chromosome X (BTAX) in mature spermatozoa of domesticated bull. We demonstrate that patterns of intranuclear location of these chromosomes in bovine spermatozoa are non-random and specific for individual chromosomes.

## 2. Materials and methods

### 2.1. Sperm nuclei preparation

Frozen semen of *Bos taurus* was provided by *Dr. P. Sutovsky (Animal Science Research Center, Division of Animal Sciences at the* University *of Missouri-Columbia*). In all experiments a combination of equal amounts of sperm cells from three different bulls was used. Sperm cells were thawed and washed three times in phosphate buffered saline (1×PBS). The cell pellet was resuspended in 1×PBS, fixed in 0.5% formaldehyde for 1 min, and then immediately washed with the excess of 1×PBS. Cellular membranes were permeabilized by incubation in 10 mM CHAPS (3-[(3-cholamidopropyl) dimethylammonio]-propane-sulfonate) in 1×PBS during 15 min at RT. Cells were washed with 1×PBS, loaded on a microscope slide in a drop of Decondensation Solution (10 mM TCEP (Tris (2-carboxyethyl) phosphine hydrochloride, SIGMA), 0.5 mg/mL Heparin sodium salt (SIGMA), 1×PBS). After incubation in a humid chamber, 30 min at RT, slides were rinsed with 1×PBS, dehydrated in 70%, 80%, 90%, and 100% ethanol series and air-dried.

### 2.2. Preparation of whole chromosome painting probes

Whole chromosome painting (WCP) probes were prepared from DNA of flow-sorted cow chromosomes 1, 29, and X kindly provided by Dr. W. Rens (Cambridge Resource Center for Comparative Genomics; Cambridge Veterinary School). Chromosome DNA was amplified and labeled with digoxigenin (DIG) by degenerate-oligonuicleotide-primed PCR (DOP PCR). Cycling parameters were the following: 3 min at 94 °C for initial denaturation; 25 cycles of 1 min at 94 °C, 1 min at 55 °C, 30 sec at 72 °C, with a 5 min final extension at 72 °C. One microgram of labeled DNA was co-precipitated with 30 micrograms of Bovine Hybloc Competitor DNA (Applied Genetics Laboratories) and 20 micrograms of Salmon Sperm DNA (Chemicon). Pellet was air-dried and dissolved in 10 microliters of hybridization mix (50% formamide, 10% dextran sulfate). Before hybridization, WCP probes were denatured at 72 °C for 10 min and preannealed for 30 min at 37 °C.

### 2.3. Fluorescence in situ hybridization

Microscope slides with cells were denatured in 70% formamide/2×SSC for 3 min at 72 °C, fixed/dehydrated in а cold ethanol series (70%, 80%, 90%, and 100%) 2 min each and air-dried. The preannealed probe was loaded onto slide, overlaid with coverslip and placed in a humid chamber for overnight hybridization at 37 °C. The hybridization was followed by two 7-min post-hybridization washes (50% formamide, 2×SSC at 45 °C), and two 7-min washes in 2×SSC at 45 °C. The slides were then incubated in the blocking solution (3% BSA, 2×SSC, 0.1% Tween-20) for 30 min at room temperature (RT). The hybridized WCP probes were detected using FITC-conjugated anti-digoxigenin, Fab fragments (Roche Diagnostics) diluted 1:200 in the blocking solution. Slides were mounted using Vectashield (Vector Labs Inc.) mounting medium with diamidino-2-phenylindole (DAPI) (Vector Labs Inc.).

### 2.4. Microscopy and image processing

**Figure 1.**
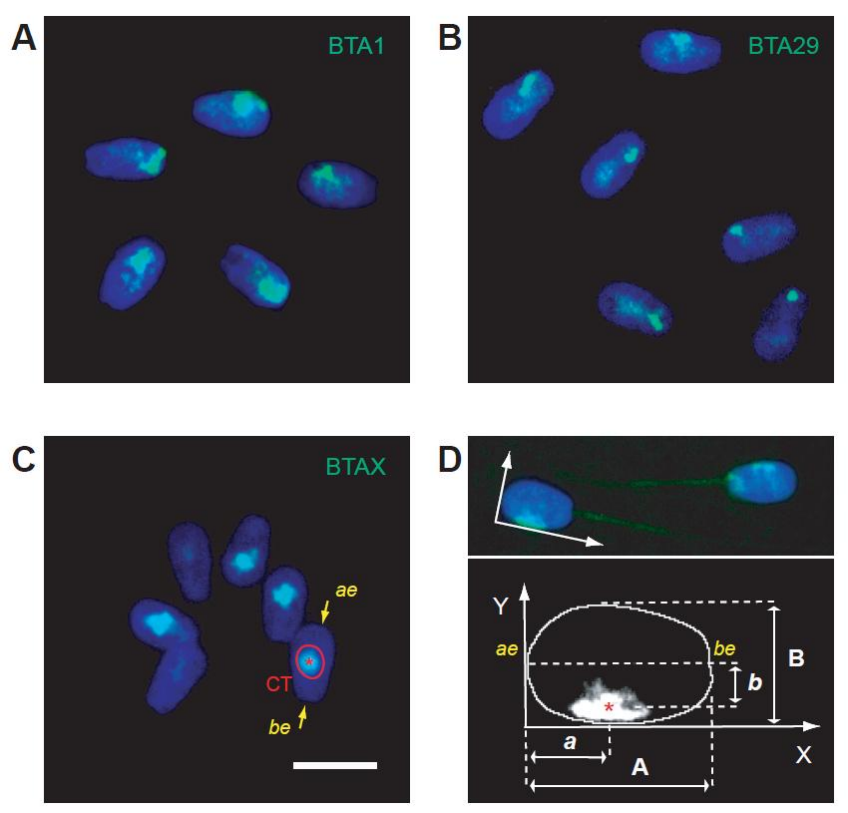
Quantification of chromosome position in *Bos taurus* spermatozoa. Typical results of fluorescence in situ hybridization (FISH) with whole chromosome painting (WCP) probes to *Bos taurus* chromosomes 1, 29 and X (BTA1- A; BTA29- B; and BTAX- C; respectively). Green—WCP hybridization signals, Blue—DAPI staining of nuclear DNA; Chromosome territory (CT) is outlined in red. Acrosomal end (ae) and basal end (be) of the sperm nucleus are pointed with arrows; D, Approach to quantification of chromosome territory (CT) position in the sperm nucleus. The acrosomal end of the sperm nucleus is placed at *x* = 0. Asterisk marks center of mass of CT. Dimensions of the nucleus and position of the CT center are quantified for each cell. Scale bar is 10 microns.

Images of the hybridized sperm samples were obtained with Leitz Ortholux fluorescent microscope equipped with 63×, 1.4 NA oil objective and fluorescein/DAPI selective band pass filter sets. Images were acquired with cooled charge coupled device (CCD) color camera (MagnaFire, Optronics Inc.) using MicroFire software (Optronics Inc.). Unbiased selection of the measured cells was assured by picking for measurements all cells with FISH signals within several arbitrary selected microscope fields. Parameters of acquisition were adjusted to avoid saturation. Images were assembled and annotated using Adobe Photoshop (Adobe Systems Inc.) and Image J 1.45 software. All measurements were performed in Sigma Scan Pro 5.0 (Systat Software Inc.) according to the scheme outlined in Figure 1D. CT center position was determined as the center of mass for an object formed by plurality of pixels that have intensity above a threshold. DAPI signal above background was used to determine the nuclear dimensions and distance of CT center to the acrosomal end and longitudinal axis of the sperm nucleus. The threshold level was determined for each cell based on the background level of the hybridization signal using DAPI based ROI.

**Figure 2.**
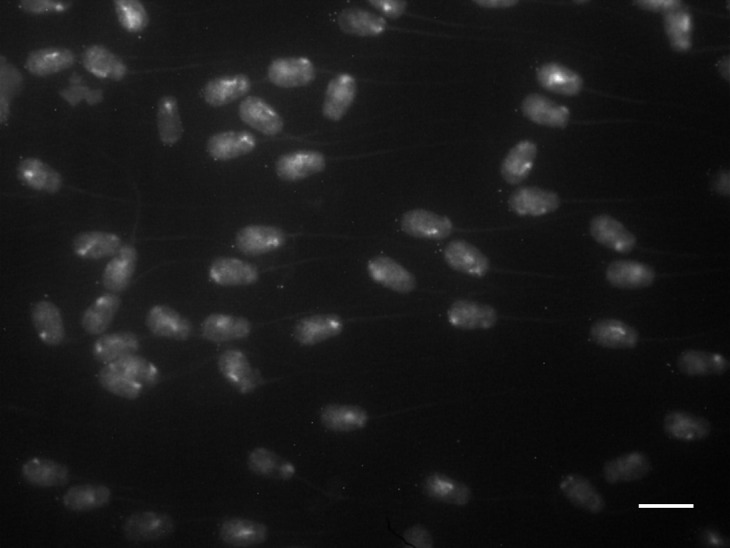
An exemplary image of *B. taurus* chromosome 29 (BTA 29) paint hybridization to bovine spermatozoa. Background hybridization signal shows that tails are preserved in most of the cells following all treatments. The tails and broadening at the acrosomal end allow easy assignment of both ends of the polarized sperm nucleus. Scale bar is 10 microns.

The applied decondensation protocol leads to very mild increase in bovine sperm size that is proportional in the axial and lateral dimensions. We did not observe any substantial differences in the shapes of sperm nuclei in the preparations. Tails were preserved in majority of the cells following the hybridization procedure (Figure 2). Most of the cell sizes of the nuclei were rather uniform and minor differences in the sizes of the nuclei were accounted for by the normalization.

**Figure 3.**
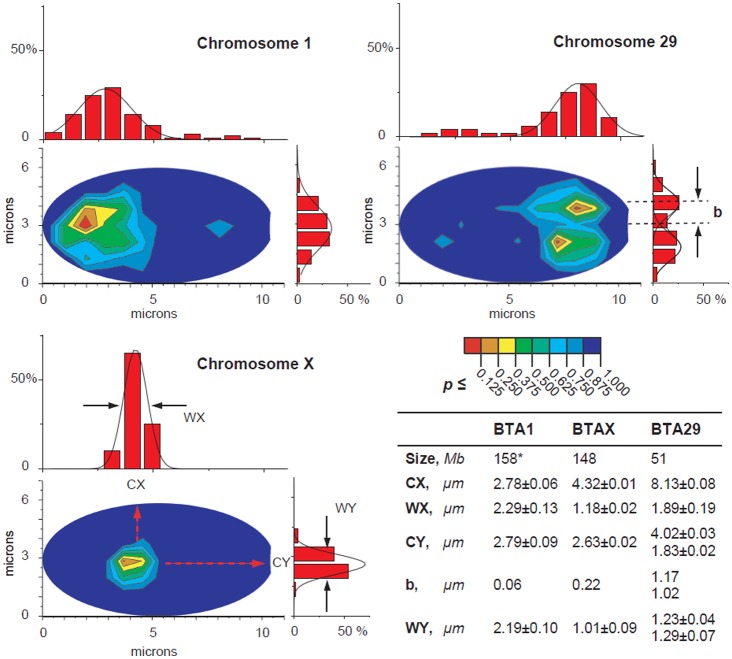
Statistical analysis of chromosome territory position. Contour graphs show color-coded probability of the CT center location for chromosomes 1, 29 and X (A, B, C, respectively) in *Bos taurus* sperm nucleus (p-value increments of 0.125). Areas of the least (*p* ≤ 0.125) and the most (0.875 ≤ *p* ≤ 1.000) probable CT locations are marked with red and navy colors, respectively. The CT center distributions along the long and short axes of the nucleus are presented as bar graphs at X and Y axes, respectively (X- and Y-histograms). Quantification results for chromosomes 1, 29 and X (BTA-1, BTA-2 and BTA-X) within an average sperm nucleus are summarized in D. Individual sperm nuclei were scaled to ellipse with short axis of 5.7 microns and long axis of 10.5 microns. CX, CY; and WX, WY are positions and FWHMs of the Gaussians fitted to the X- and Y-histograms, respectively; b is the distance of the of CT center from the central axis of the nucleus: b = |2.85−CX|.

Contour plots and frequency distribution histograms were generated using Origin 8.6 (OriginLab Corp.) software. The preferable positions of the CT centers were determined by Gaussian approximation of frequency distribution histograms using Origin 8.6 (OriginLab Corp.) software (See Figure 3 for details). Centers (Cx and Cy) and FWHM (Wx and Wy) of the fitted Gaussians were determined and used in further calculations.

## 3. Results

Bovine spermatozoa are elongated cells, flattened in a dorsoventral direction (“paddle-shaped”) and polarized in the anteroposterior direction due to the presence of an acrosome at the slightly broadened anterior pole and tail at the posterior pole of the cell (Figure 1). Applied method of decondensation did not affect the morphology of spermatozoa, so polarity of native cells could be easily seen in cells in the preparations (Figure 2), providing for clear-cut detection of the location of the hybridization signals.

To map the intranuclear localizations of ВТA1, ВТA29 and ВТAX, we applied the approach recently developed by us for human spermatozoa [14]. After performing FISH and imaging the preparations, nuclei of the sperm cells were approximated by an ellipse based on the DAPI signal. The origin of coordinates was placed at the acrosomal end as opposite to basal end marked by the sperm tail, which was preserved in the majority of cells in the preparations. WCP probes yield FISH signals corresponding to the whole chromosome territory (CT). Therefore, similarly to Foster et al. [18], we used geometrical center of FISH signal (CT center) as a characteristic of the chromosome position within the sperm nucleus. Intranuclear chromosome positions were quantified by measuring longitudinal (in the anterior/posterior direction) and lateral (distance from the long nuclear axis) coordinates of the CT centers (Figure 1D). To account for the differences in nuclear sizes, the measured longitudinal (x) and lateral (y) coordinates of the CT centers for each nucleus were normalized to the average dimensions of a sperm nucleus in the preparation (W × L = 10.5 × 5.7 microns). Hybridization signals of at least one hundred cells were examined for each of chromosome 1 (BTA1, n = 102), chromosome 29 (BTA29, n = 132) and chromosome X (BTAX, n = 100), which roughly corresponds to 30–40 cells from each animal. No differences were observed between individual hybridization experiments. Data from single hybridization for each of the chromosomes is presented. The measured coordinates of the CT centers were used to color-code the frequency of chromosome localization in particular region of the nucleus: the nuclear area was subdivided based on the series of ranges of *p* values for probability of the CT center presence in particular nuclear location with increments of 0.125. The resulting contour plots allow convenient statistical visualization of the characteristic patterns of the intranuclear distribution of geometrical CT centers within an average *Bos taurus* sperm nucleus (Figure 3).

As seen in Figure 3, the contour plots for ВТА1 and ВTAX contain single most probable area of CT center location close to the long axis of the nucleus, which is indicative of the tendency of the internal location of these chromosomes.

The contour plot for BTA29 contains two most probable regions almost symmetrical about the long nuclear axis. The observed separation of the most probable area from the long axis of the sperm nucleus and extended lateral dimensions of the frequency plot are indicative of the tendency of peripheral location of BTA29. The existence of two symmetrical most preferable areas is a consequence of the central plane of symmetry (2-fold rotational symmetry) of the flattened nuclei of bull spermatozoa, which leads to two most likely modes of their deposition on the microscope slide: one or another flat side down. Such “flip-flop” effect of sperm cells deposition onto the slide can be only noticed in case of peripheral chromosome location, and would not be evident for the centrally located chromosomes.

Cross-sections of the plots both in longitudinal and lateral directions are presented as frequency distribution histograms along x-axis (“X-histograms”) and y-axis (“Y-histograms”) of the nucleus (Figure 3).

X-histograms show the longitudinal distribution of the CT centers of BTA1, BTA29 and BTAX in anterior/posterior direction of *Bos taurus* sperm nuclei. BTA1 is located in the apical (acrosomal) one-third of the sperm nucleus, BTAX in the medial third, BTA29 in the basal third. Thus, the largest (BTA1) and the smallest (BTA29) autosomes occupy the opposite ends of the elongated sperm nuclei, and BTAX is localized almost centrally (slightly shifted to the acrosomal end).

Y-histograms (Figure 3) show the lateral distribution of the CT centers along the short axis of bovine spermatozoa. Y-histograms contain single peaks of the preferred positioning for BTA1 and BTAX, while the histograms for BTA29 show two symmetrical peaks with almost equal heights. The observed bimodality very likely arises from the two equiprobable modes of deposition of spermatozoa on the slide discussed above.

As seen in the x-histograms, distribution for BTAX nuclear localization is much narrower in comparison with that of BTA1 and BTA29, which suggests a more constrained location of BTAX as compared to BTA1, which comprise roughly the same amount of DNA (Figure 3 and Table therein).

## 4. Discussion

In the presented study, we mapped preferential intranuclear locations of X chromosome and two autosomes (the largest and the smallest within the karyotype) in bovine spermatozoa by applying a combination of FISH and statistical image analysis that was developed for human spermatozoa [14].

Previous studies of chromosome positioning in mammalian spermatozoa utilized various approaches for characterizing chromosome position within the nucleus. The area of the elongated sperm nucleus was divided into sectors, “subacrosomal, equatorial, basal” [26], “regions (I–IV)” [10], “apical, medial, basal” [13], or into several concentric zones or “shells” [4],[15], and the number of FISH signals in each zone was scored. Alternatively, to determine the chromosome position, the normalized distances from FISH signal to either the tail attachment spot [10] or the nearest peripheral edge [19] were computed.

The approach used in the present study represents a two-step procedure, when the center of CT in the sperm nucleus is first mapped, and then the distribution of the CT position is plotted based on the repeated measurements in a number of cells.

The choice of the analysis approach may depend on the shape of the sperm cells. Dividing sperm nucleus into concentric circles allows assessing central or peripheral tendency in chromosome location under assumption of radial symmetry of the nucleus. However, the frequency of chromosome location in each of the shells shall be normalized to the relative shell volume or area. When radius of the nucleus is divided into three equal portions, the areas or volumes of the central and peripheral shells differ 8 or 27 times. The normalization and calculation of the corresponding probabilities becomes even more complex when CT spans more than one shell. The presently used analysis does not directly account for the shape of the CT, but represents an easy to implement approach for sensing major tendencies in nuclear CT positioning in elongated cells.

Graphical representation of the likelihood of the CT center position by means of the color-coded contour plots (Figure 3) was used to reveal the characteristic patterns of the CT center distribution and simultaneous assessment of regularities in both longitudinal (anterior/posterior) and lateral (internal/peripheral) intranuclear positioning of the chromosomes.

We show that all three studied *Bos taurus* chromosomes demonstrate non-random preferential lateral and longitudinal intranuclear positioning as evident both from the frequency plots and histograms. Our findings are in agreement with the previous chromosome positioning studies on mammalian spermatozoa [10]–[16],[18],[19].

The largest (BTA1) and the smallest (BTA29) bovine autosomes tend to be located at the opposite ends of the sperm nuclei in longitudinal direction: BTA1 was preferentially localized in the acrosomal part, while BTA29 was preferentially found in the basal part of sperm nucleus. Laterally, BTA29 was located towards nuclear periphery as compared with the more internal position of BTA1.

BTA X is 148 Mb in size and contains 1643 genes, which is comparable to the size and gene content of BTA1 (158 Mb and 1267, respectively). Our results show that BTAX is located in the medial third of the nucleus, slightly shifted towards acrosomal end (4.32 µm peak out of 10.5 µm). Similar sex chromosome locations were found in pigs [19], humans, [14], mice [23] and it was suggested that sex chromosomes should be the last ones to be remodeled by contacting the components of egg cytoplasm upon the inclusion of the sperm nucleus into egg [19]. According to the opposite point of view, sex chromosomes are supposed to be the first chromosomes affected by egg environment [2]. This point of view is based on studies of X chromosome location in spermatozoa of non-placental mammals: marsupials [1],[2] and monotremes [2]. Despite the disagreement on the suggested timing of sex chromosome remodeling, all authors concurred in that intranuclear localization of sperm chromosomes might influence the order of chromosome processing and activation during natural or artificial fertilization [2],[18]. Taking into account that sex chromosomes are enriched for genes related to sex differentiation and reproduction, sex chromosome positions in male gametes could be critical for the process of fertilization.

Another noticeable feature of BTA X nuclear localization was much narrower distribution of CT center signals in comparison with those for BTA1 and BTA29 (Figure 3). A mixture of sperm cells from three individual animals was used in our experiments. Therefore, we did not specifically address differences in sperm nuclear organization between individual bulls. However, our data suggest that if any such differences are present, their degree is substantially less for chromosome X, than for chromosomes 1 and 29.

Similar characteristics of sex chromosomes territories were reported previously for other animals. It was shown that X and Y chromosome territories are organized in compact spherical structures in pigs [19], mice [23], and humans [27], indicative of sex chromosome specific chromatin modifications and its denser packaging. In mammalian male germ cells, X chromosome is involved in process called sex chromosome inactivation, which begins in meiosis, persists throughout spermatogenesis into mature sperm and possibly provides for imprinted paternal X-chromosome inactivation in female embryos [23],[27]. It is accompanied by chromatin silencing through binding to conserved heterochromatin proteins CBX1 (HP1β), CBX3 (HP1γ) and epigenetic modification of chromatin such as trimethylation of histone H3 lysine 9 (H3K9me3). Altogether, that leads to formation of distinctive inert nuclear compartment juxtaposed to the chromocenter(s) [23],[27].

Ribosomal and centromeric sequences have been shown to be confined to the equatorial region and have postacrosomal location in bovine sperm nuclei [28]. As the postacrosomal region decondenses first during fertilization, it was suggested that ribosomal and centromeric sequences should be decondensed early. Early release of centromeric regions may allow swift attachment of chromosomes to microtubules of the spindle via kinetochore after dismantling the sperm head into individual chromosomes [28]. In such a case, X chromosome juxtaposed to the chromocenter would be also decondensed early after fertilization. It is of interest to see whether X chromosome territory is adjacent to the chromocenter in bovine spermatozoa.

Previous studies where the chromosome positioning in spermatozoa was analyzed in relation to their size did not provide consistent results. No significant correlation between chromosome size and longitudinal or lateral nuclear position was found in pigs [19] and humans [14]. At the same time, strong association between chromosome size and the radial distance of the chromosome from the nuclear center has been reported in humans [12].

It should be noted that the precision of CT location measurements may be affected by cell-to-cell differences in the shape of the chromosome territory. However, in the compact sperm nucleus the dimensions of chromosome territory are unlikely to be affected by variable chromatin condensation and depend more directly on the chromosome size. Accordingly, it is expected that for larger chromosomes, contour plots for distribution of the CT position will cover a bigger nuclear portion. This tendency is evident from the shape/size of contour plots for chromosomes 1 and 29 (Figure 3). Notably, both longitudinal and lateral radial locations of BTAX were more constrained in comparison with the BTA1, which is close to BTAX in size.

## 5. Conclusion

This study reports preferred locations of chromosomes in *Bos taurus* spermatozoa thus providing another evidence of non-random chromatin architecture in mammalian male gametes. In line with earlier reports, our results suggest the existence of the sperm chromatin positioning factors specific to individual chromosomes.

Upon fertilization, sperm chromatin is decondensed by the action of oocyte factors. The order in which paternal chromosomes and their sub-regions contact with oocyte cytoplasm can influence dynamics of substitution of sperm specific protamines to histones and expression profile of the paternal genome. Therefore, chromosome positioning in the sperm nucleus may represent yet another aspect of epigenetic information that is transferred between generations.

Further studies of the chromatin remodeling events at early stages of embryogenesis will be essential for understanding the functional aspects of non-random genome architecture in spermatozoa.
